# Diagnostic efficiency on ultrasound shear wave elastography in evaluation of steatosis severity for non-alcoholic fatty liver disease: a rat model

**DOI:** 10.1186/s40001-023-01042-5

**Published:** 2023-02-11

**Authors:** Yuhui Wu, Qianjiao Liu, Yan Wang, Fangyan Li, Lawrence Wing-Chi Chan, Yong Wen, Fan Yang, Yining Xiang, Qinghong Duan, Peng Luo, Pinggui Lei

**Affiliations:** 1 Department of Radiology, the Affiliated Hospital of Guizhou Medical University, No. 28 Guiyi Street, Yunyan District, Guiyang, 550004 Guizhou China; 2 School of Public Health, Guizhou Medical University, Guiyang, Guizhou China; 3 Department of Health Technology and Informatics, The Hong Kong Polytechnic University, Hong Kong, SAR China; 4 School of Biology & Engineering, Guizhou Medical University, Guiyang,, Guizhou China; 5 Department of Pathology, The Affiliated Hospital of Guizhou Medical University, Guiyang, Guizhou China

**Keywords:** Shear wave elastography, Non-alcoholic fatty liver disease, Hepatic steatosis, Methionine choline deficiency, Fat quantification

## Abstract

**Background:**

The pathological feature of steatosis affects the elasticity values measured by shear wave elastography (SWE) is still controversial in non-alcoholic fatty liver disease (NAFLD). The aim of this study is to demonstrate the influence of steatosis on liver stiffness measured by SWE on a rat model with NAFLD and analyze feasibility of SWE for grading steatosis in absence of fibrosis.

**Methods:**

Sixty-six rats were fed with methionine choline deficient diet or standard diet to produce various stages of steatosis; 48 rats were available for final analysis. Rats underwent abdominal ultrasound SWE examination and pathological assessment. Liver histopathology was analyzed to assess the degree of steatosis, inflammation, ballooning, and fibrosis according to the non-alcoholic fatty liver disease activity score. The diagnostic performance of SWE for differentiating steatosis stages was estimated according to the receiver operating characteristic (ROC) curve. Decision curve analysis (DCA) was conducted to determine clinical usefulness and the areas under DCA (AUDCAs) calculated.

**Results:**

In multivariate analysis, steatosis was an independent factor affecting the mean elastic modules (*B* = 1.558, *P* < 0.001), but not inflammation (*B* =  − 0.031, *P* = 0.920) and ballooning (*B* = 0.216, *P* = 0.458). After adjusting for inflammation and ballooning, the AUROC of the mean elasticity for identifying *S* ≥ *S*1 was 0.956 (95%CI: 0.872–0.998) and the AUDCA, 0.621. The AUROC for distinguishing *S* ≥ *S*2 and *S* = *S*3 was 0.987 (95%CI: 0.951–1.000) and 0.920 (95%CI: 0.816–0.986) and the AUDCA was 0.506 and 0.256, respectively.

**Conclusions:**

Steatosis is associated with liver stiffness and SWE may have the feasibility to be introduced as an assistive technology in grading steatosis for patients with NAFLD in absence of fibrosis.

## Background

Non-alcoholic fatty liver disease (NAFLD) is a chronic disease characterized by hepatic fat accumulation combined with underlying metabolic dysregulation, mainly encompassing hepatic steatosis, non-alcoholic steatohepatitis (NASH), non-alcoholic cirrhosis, and even hepatocellular carcinoma [1, 2]. It is rapidly becoming one of the most common liver diseases, with a 20–30% incidence in Western countries [3].

Liver biopsy (LB) is regarded as the gold standard for assessing disease severity [4, 5]. However, due to tissue invasion, sampling error, and the associated complications [6], imaging methods are increasingly being favored for the non-invasive evaluation of fatty liver. B-Mode Ultrasound (US) based on grayscale images is the most common method for evaluating fatty liver due to its low cost, safety, and availability. Hepatic steatosis is usually graded by various US features, including liver brightness, the hepatorenal ratio, and vessel blurring [7, 8]. However, the sensitivity of US B-mode imaging for detecting mild steatosis (fat content > 5%) is reportedly between 60.9% and 65% [9], and it does not allow quantitative evaluation. Although the controlled attenuation parameter (CAP) based on Fibroscan can accurately detect and quantify liver steatosis (> 10%), a meta-analysis showed that overlapping boundaries limit CAP’s clinical value in distinguishing between mild and moderate steatosis [10, 11]. Liver ultrasound attenuation (LiSA) is a new technique similar to CAP, but it may not accurately reflect the actual performance of LiSA tested in LB-validated patients [12]. In contrast, Sound Speed Estimation could detect and grade hepatic steatosis with a sensitivity of 80% and specificity of 85.7% in a small pilot study, whereas further research in a larger sample of patients with NAFLD is needed [13].

Although CT is more effective in assessing fatty liver, it is less accurate for mild to moderate hepatic steatosis, and involves radiation exposure [14]. Magnetic resonance spectroscopy is a non-invasive method for the quantification of liver fat with high sensitivity and specificity; nevertheless, at present it is mainly used as a research tool [15].

Ultrasound elastography techniques, like transient elastography (TE), shear wave elastography (SWE), or acoustic radiation force impulse(ARFI), based on shear wave generation, aim to measure elasticity [16]. TE showed a great performance when identifying and staging fibrosis in NAFLD, but steatosis or inflammatory activity may influence the accuracy of liver stiffness measurements (LSMs) in predicting fibrosis [17–19]. A large-scale meta-analysis has shown that 2D-SWE is superior to TE in diagnosing significant fibrosis and cirrhosis [20]. However, whether steatosis affects the elasticity values measured by SWE is still controversial [21]. Some studies found no correlation between steatosis and SWE measurements based on multivariate analysis [22, 23], whereas others have shown increasing liver viscoelasticity with the presence of steatosis [24].

Hence, we performed an experimental study in rats that had undergone SWE examinations, using liver biopsy as reference standard. This study aimed to investigate the correlation between hepatic steatosis and elastic modulus values measured by SWE and discuss feasibility of SWE for grading steatosis in the absence of fibrosis in NAFLD.

## Methods

This study was approved by the animal ethics committee of our university in agreement with committee guidelines.

### Animal model construction

All animals were provided by the animal experiment center of our university and fed in the animal room of our university. Eight-week-old male Sprague–Dawley rats (*n* = 66) were randomly assigned to two groups after adaptation feeding for a week: A control group (*n* = 10) was fed with standard diet, and the experimental group (*n* = 56) fed with a methionine choline deficient (MCD) diet. The experimental group underwent US-SWE on the 2nd week (*n* = 8), 4th week (*n* = 8), 6th week (*n* = 10), 8th week (*n* = 10), 10th week (*n* = 10), and 12th week (*n* = 10) to measure various levels of steatosis severity. Similarly, the control group was also studied at 4th week (*n* = 2), 6th week (*n* = 2), 8th week (*n* = 2), 10th week (*n* = 2), and 12th week (*n* = 2). During the experimental process, rats were had ad libitum access to food and water.

### US-SWE examination

**Fig. 1:**
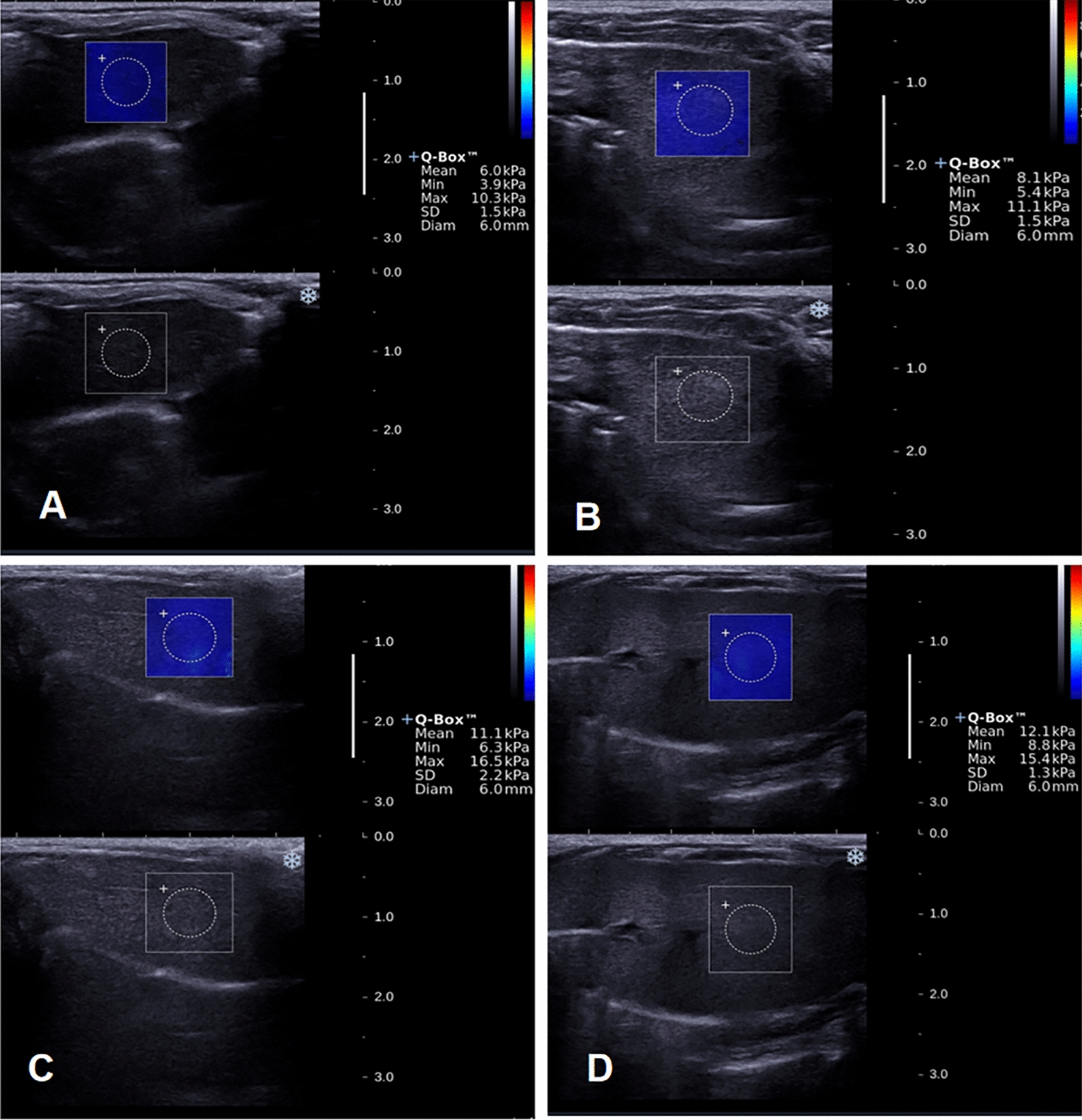
US-SWE diagrams of steatosis with different degrees in NAFLD. **A** Normal **B** Mild steatosis **C** Moderate steatosis **D** Severe steatosis. Mean, minimum, maximum elastic modulus, and SD values of various degrees are illustrated in the corresponding graphs

Animals’ skin was prepared for a better window. All animals were placed on an examination bed in a supine position under anesthesia. The US-SWE device equipped with a Supersonic Imagine Aixplorer with a linear-array transducer and frequency of 4–15 MHz was employed. First, the liver condition, including size, morphology, and echogenicity, was observed in 2D grayscale images, and then the probe was gently fixed on the rats’ skin. Subsequently, we switched to SWE mode to display the sampling frame with the appropriate liver parenchyma as examination site, avoiding large vessels, ducts, and inter-lobar fissures [25]. The tissue stiffness in the SWE image ranges from dark blue, indicating the lowest stiffness level (0 kPa), to red, indicating the highest level (up to 70 kPa). Color filling uniformity was > 90% in the sampling frame, considered as successful sampling. After a few seconds, when the SWE image stabilizes, it is frozen and stored. The minimum, mean, maximum elastic modulus, and standard deviation (SD) in the sampling frame were automatically calculated by the Q-box system using regions of interest (ROI) of 6 mm in diameter approximately (Fig. 1). LSM was performed three times for each subject, and the average of those three acquisitions in kilopascals (kPa) was used for statistical analysis. The above operations were referred to recommendation by EFSUMB Guidelines [21].

### Histopathological assessment

After the examination, the rats were sacrificed via intraperitoneal injection of overdose anesthesia, and the liver was dissected and completely removed. The liver tissue of each animal was stained by hematoxylin and eosin and Masson’s trichrome. An experienced pathologist blinded to elastic module values and diet reviewed the biopsy specimens and scored the histologic findings. The scoring system comprised histological features evaluated according to the semi-quantitative NAFLD activity score [26]: Steatosis (0–3), lobular inflammation (0–3), hepatocellular ballooning (0–2), and fibrosis stages (0–4). Steatosis stages were determined by the percentage of hepatocytes containing intracytoplasmic fat droplets. The pathological findings of animals were classified into normal, mild steatosis, moderate steatosis, and severe steatosis groups [27]. The staging characteristics of histological findings are shown in Table 1.

**Table 1:** Stages of histological features with NAFLD

Histological features	Stages				
	0	1	2	3	4
Steatosis	< 5%	5–33%	34–66%	> 66%	–
Lobular inflammation	None	1–2foci/200 × field	2–4foci/200 × field	> 4foci/200 × field	–
Hepatocyte ballooning	None	few balloon cells	Many balloon cells	**–**	**–**
Fibrosis	None	Perisinusoidal /periportal	Perisinusoidal and portal/periportal	Bridging fibrosis	Cirrhosis

### Statistical analysis

Continuous variables are expressed as median and interquartile range (IQR). The correlation between histopathologic findings (steatosis, inflammation, and ballooning) and four parameters from US-SWE (mean, maximum, minimum elastic modulus values, and SD) was investigated using Spearman's rank correlation analysis. Multivariate regression analysis was performed to test the relationship between mean elastic value and pathological features. A post hoc Games−Howell test after one-way analysis of variance was used to compare differences among groups of histopathologic findings. The predictive performance for differentiating steatosis stages was estimated according to the area under ROC curve (AUROC). The optimal threshold was determined by maximizing the Youden index (equal to the sum of sensitivity and specificity minus 1), and also the sensitivity, specificity, positive predictive values (PPVs), and negative predictive values (NPVs) associated with 95% confidence interval (95%CI) were calculated. Decision curve analysis (DCA) was conducted to determine clinical usefulness and the area under decision curve (AUDCA) calculated. Data were analyzed using SPSS, Stata, the statistical software packages R (http://www.R-project.org, The R Foundation), and EmpowerStats (http://www.empowerstats.com, X&Y Solutions, Inc, Boston, MA). P < 0.05 indicates statistical significance.

## Results

### Animal model and histological findings

A total of 66 rats were used in this study, of which 60 survived and 6 in the MCD group were naturally sacrificed. Specifically, three rats died in the 6th week, 8th week, and 10th week, respectively; the other three rats died in the 12th week, which may be due to intolerance to anesthetics and abdominal effusion. Meanwhile, 10 rats in the control group survived. Given that the presence of fibrosis might be a significant confounding factor, rats with fibrosis (*n* = 12) were excluded in this study. Ultimately, 48 rats were eligible for the final analysis. All enrolled animals were classified into a normal (14/48, 29.1%), mild steatosis (5/48, 10.4%), moderate steatosis (6/48, 12.5%), and severe steatosis (23/48, 47.9%) groups.

**Fig. 2:**
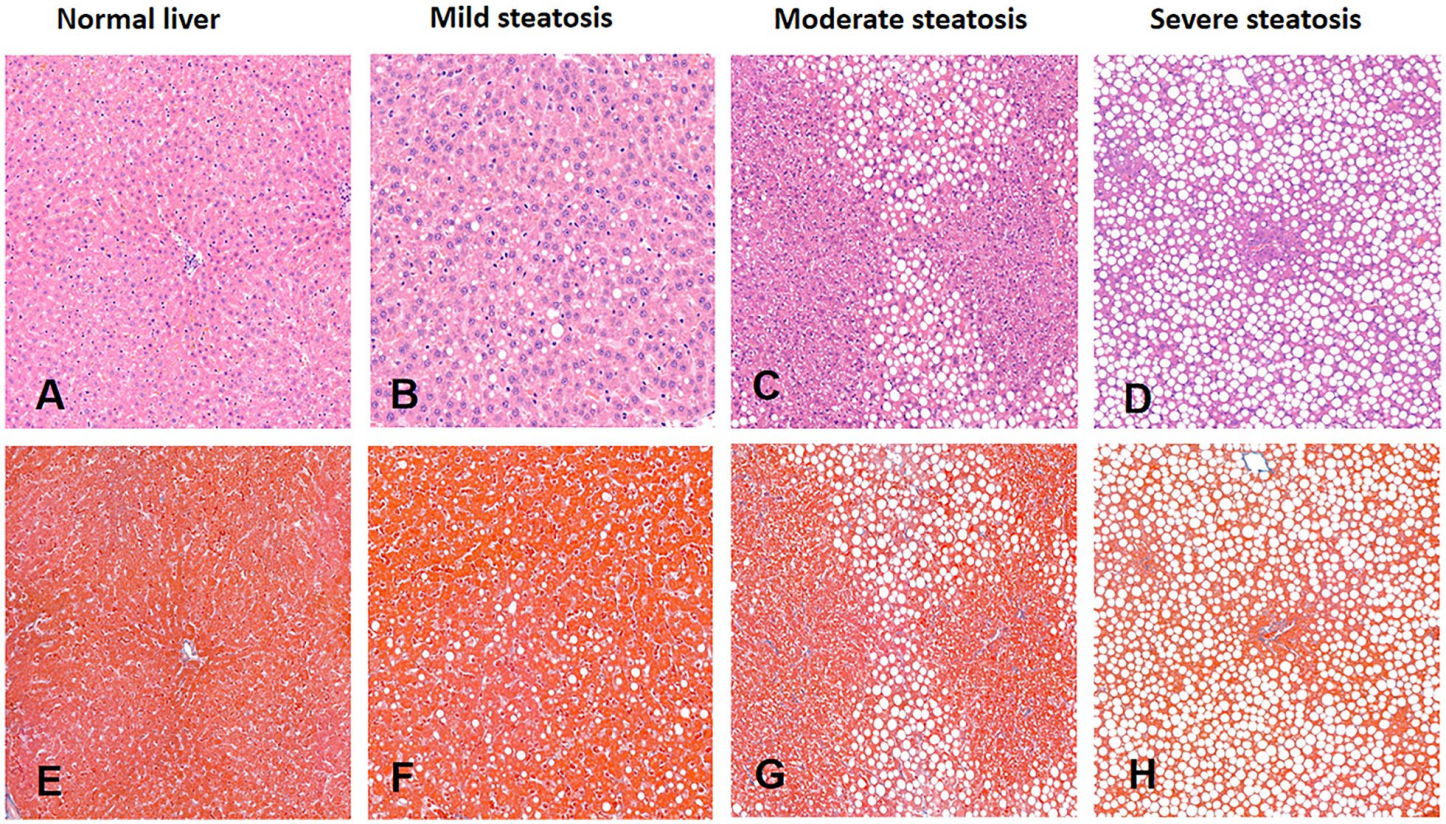
Histological NAFLD sections with different degrees of steatosis. **A** and **E** Hematoxylin and eosin staining and masson trichrome staining demonstrating the pathological features of steatosis in normal liver, **B** and **F** mild fatty liver, **C** and **G** moderate liver, **D** and **H** severe fatty liver

The distribution of pathological features in NAFLD at different time points is summarized in Table 2, and histological sections of NAFLD with various degrees of steatosis are displayed in Fig. 2. Under the microscope, the structure of the lobular liver in the control group was normal, with clear hepatic lamina structure and sinusoids arranged in a spoked shape. Simultaneously, the nucleus was in the cell center, without fat droplets within the cell. The liver of rats in the experimental group showed multiple manifestations of different degrees of steatosis: For mild steatosis: Scattered fat droplets in hepatocytes, present hepatic lamina and sinusoids, and centered nucleus. Moderate steatosis: Many fat droplets in hepatocytes and some compressed and displaced nuclei. Severe steatosis: Liver cells were filled with numerous fat droplets, compressed and displaced nuclei, disorganized liver structure, and lost hepatic blood sinus structure.

**Table 2:** Distribution of pathological features in NAFLD at different time points

Rearing time	Histological findings			
	Steatosis	Inflammation	Ballooning	Fibrosis
	S0/S1/S2/S3	I0/I1/I2/I3	B0/B1/B2	F0/F1/F2/F3/F4
2w (*n* = 8)	6/2/0/0	0/3/5/0	5/3/0	8/0/0/0/0
4w (*n* = 10)	2/2/2/4	2/4/2/2	9/1/0	10/0/0/0/0
6w (*n* = 11)	2/2/1/6	6/5/0/0	7/1/3	10/0/1/0/0
8w (*n* = 11)	1/0/3/7	2/9/0/0	5/1/5	11/0/0/0/0
10w (*n* = 11)	1/0/1/9	2/8/1/0	10/1/0	7/3/1/0/0
12w (*n* = 9)	2/3/1/3	1/4/0/4	7/1/1	2/3/0/2/2

Data represent the number of pathologic features

### Histopathological features and mean elastic value

**Fig. 3:**
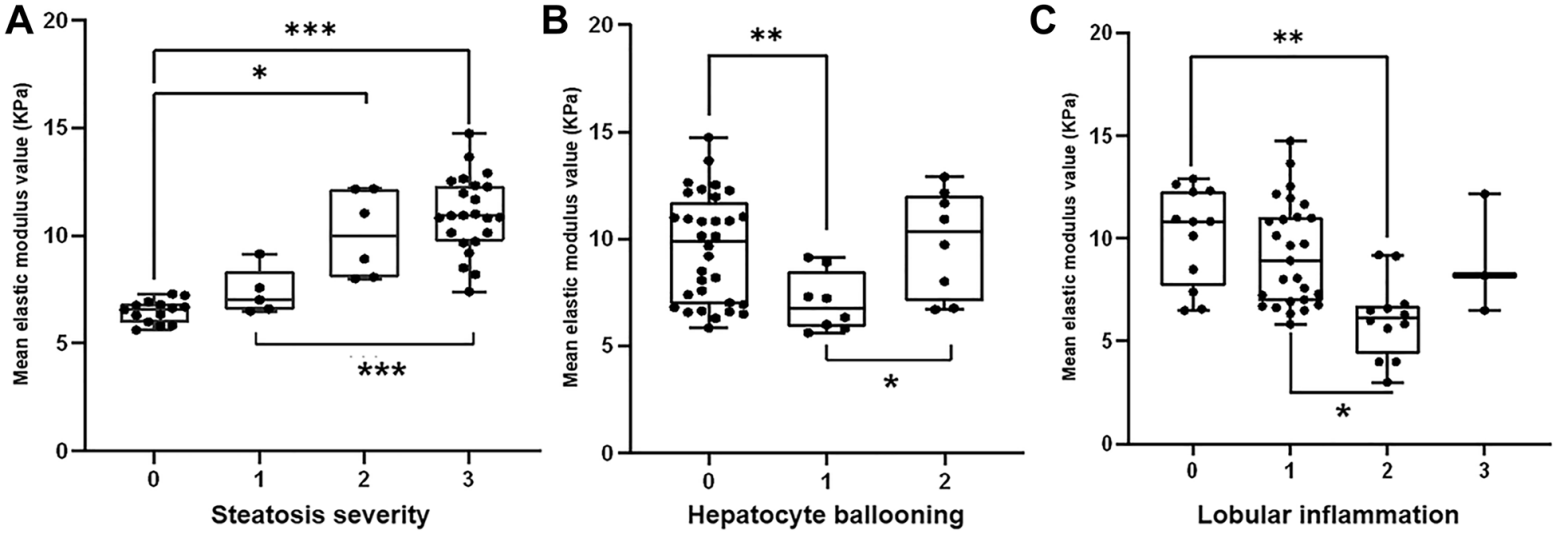
Distribution of elasticity values in pathological features. The boundaries in box plots show upper and lower quartiles, and the lines in the box the median. Asterisks (*) indicate a Games–Howell test with significant differences

The median of the mean elastic modulus values of normal, mild, moderate, and severe groups were 6.60 kPa (IQR: 6.08, 6.79), 7.02 kPa (IQR: 6.60, 7.58), 9.98 kPa (IQR: 8.28, 11.88), and 10.92 kPa (IQR: 9.93, 12.30), respectively. The distribution of mean modulus values according to histological features is illustrated in box plots (Fig. 3). The mean elastic modulus steadily increased with steatosis stages. As for lobular inflammation, the mean elastic modulus value was highest in grade 0, initially decreased with inflammation degree, reached the lowest value in grade 2, and increased slightly in grade 3. Regarding hepatocellular ballooning, there were significant differences in mean elastic modulus between grades 0 and 2, as well as between grades 1 and 2.

### Correlation and multivariate regression analysis

**Fig. 4:**
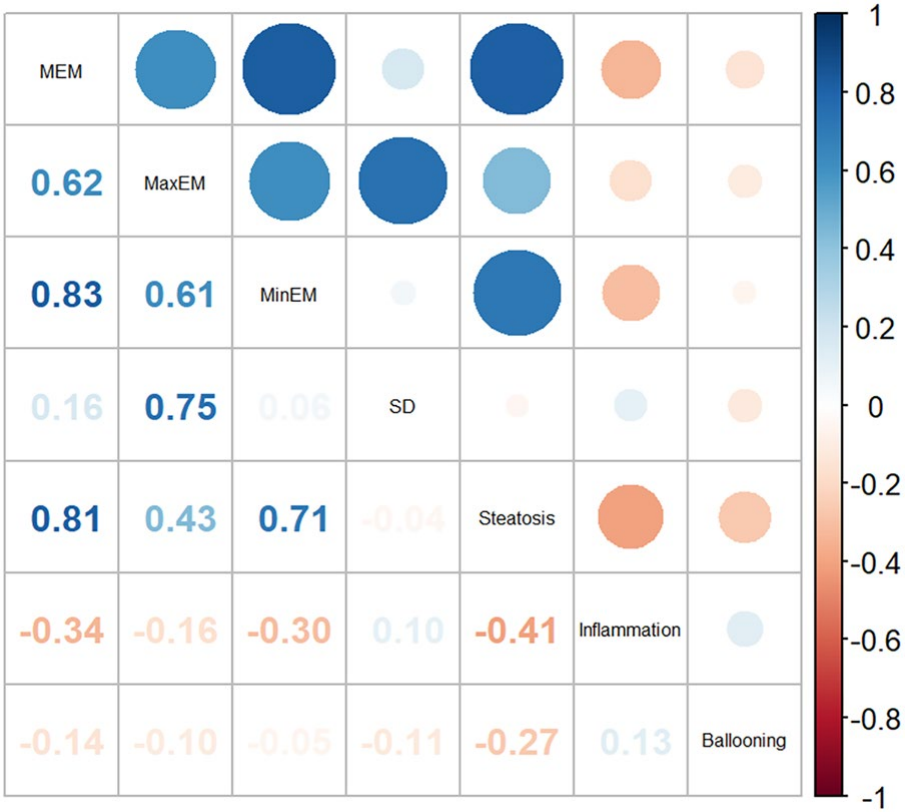
Correlation between elasticity value and pathological features. The red circle represents a negative correlation, the blue circle a positive correlation; the value in the lower left corner represents the correlation coefficient in Spearman's rank correlation analysis. *MEM* mean elastic modulus value, *MaxEM* maximum elastic modulus value, *MinEM* minimum elastic modulus value, *SD* standard deviation.

The correlation between elastic modulus values and histological findings is shown in Fig. 4. Steatosis (*ρ* = 0.81, *P* < 0.001) and lobular inflammation (*ρ* =  − 0.335, *P* = 0.03) were significantly correlated with liver mean elastic value, but hepatocyte ballooning was not significantly correlated with elastic modulus value (*ρ* =  − 0.143, *P* = 0.333). In multivariate analysis, steatosis was an independent factor affecting the mean elastic modules (*B* = 1.558, *P* < 0.001), whereas inflammation (*B* =  − 0.031, *P* = 0.920) or ballooning (*B* = 0.216, *P* = 0.458) was not. Table 3 shows increased elasticity of different degrees of steatosis compared to the normal status. The mean elastic modules in mild, moderate, and severe steatosis groups were higher [0.88 kPa (95%CI: − 0.64 to 2.40, *P* = 0.261), 3.57 kPa (95%CI: 2.15–4.99, *P* < 0.001), and 4.51 kPa (95%CI: 3.52–5.50, *P* < 0.001)] than those in the normal group. After adjusting for inflammation, ballooning, and both, the mean elastic modulus values changed accordingly, but the mean elastic modulus value with moderate or severe steatosis was still higher than that in the normal group (*P* < 0.001).

**Table 3:** Association between NAFLD steatosis stages and mean elastic values adjusted for other pathological features

Parameters	Non-adjusted	Adjust I	Adjust II	Adjust III
S0	Reference	Reference	Reference	Reference
S1	0.88 (− 0.64, 2.40) kPa 0.261	0.96 (− 0.60, 2.52) kPa 0.235	0.94 (− 0.66, 2.54) kPa 0.255	0.97 (− 0.67, 2.61) kPa 0.251
S2	3.57 (2.15, 4.99) kPa < 0.001	3.42 (1.85, 4.99) kPa < 0.001	3.54 (2.04, 5.04) kPa < 0.001	3.40 (1.76, 5.05) kPa < 0.001
S3	4.51 (3.52, 5.50) kPa < 0.001	4.49 (3.35, 5.64) kPa < 0.001	4.53 (3.37, 5.69) kPa < 0.001	4.50 (3.22, 5.78) kPa < 0.001

Data presented by β (95%CI) *P* value; Independent variable: Steatosis grades; Dependent variable: Mean elastic modules

Non-adjusted model: None

Adjust I: Model adjusted for inflammation of histological features in rat NAFLD model

Adjust II: Model adjusted for ballooning of histological features in rat NAFLD model

Adjust III: Model adjusted for inflammation and ballooning of histological features in rat

### The performance of SWE on evaluation of NAFLD steatosis

**Fig. 5:**
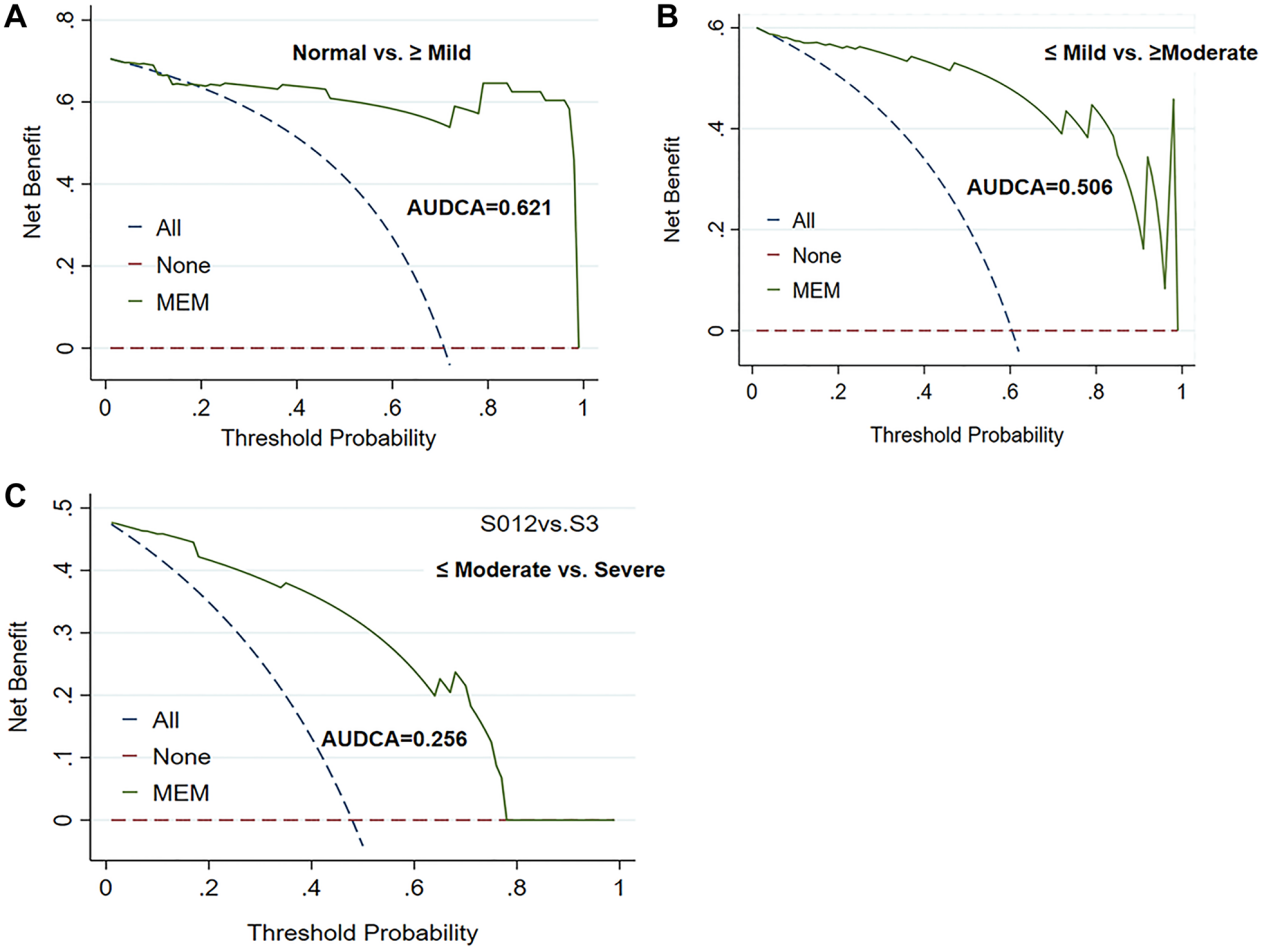
Decision curve analysis (DCA) of mean elastic modulus value of US-SWE. **A** AUDCA for identifying normal liver and mild steatosis or higher severity was 0.621. **B** AUDCA for distinguishing normal or mild steatosis and moderate or higher severity was 0.506. **C** AUDCA for differentiating severe steatosis and moderate or lower severity was 0.256. *MEM* mean elastic modulus value, *AUDCA* area under decision curve analysis

The performance of mean elastic modules in grading the steatosis was analyzed by ROC. Our results showed that the AUROC of the mean elastic modulus model for identifying ≥ *S*1 was 0.964 (95%CI: 0.917–1.000), for distinguishing ≥ *S*2 it was 0.987 (95%CI: 0.963–1.000), and for differentiating *S* = 3 it was 0.904 (95%CI: 0.812–0.996). The corresponding optimal threshold, sensitivity, specificity, PPVs, and NPVs are summarized in Table 4. After adjusting for inflammation and ballooning, the AUROCs for mean elastic modulus for the prediction of steatosis stages were > 0.90 regardless of the presence or absence of inflammation and ballooning. The DCA of mean elastic modulus values is presented in Fig. 5. The AUDCA for identifying ≥ *S*1 was 0.621, for distinguishing ≥ *S*2 it was 0.506, and for differentiating *S* = 3 it was 0.256.

**Table 4:** The predictive efficiency of SWE for NAFLD assessment

Steatosis	*S* ≥ *S*1	*S* ≥ *S*2	*S* = *S*3
AUC (95%CI)	0.964 (0.917, 1.000)	0.987 (0.963, 1.000)	0.904 (0.812, 0.996)
AUC* (95%CI)	0.956 (0.872, 0.998)	0.987 (0.951, 1.000)	0.920 (0.816, 0.986)
Cutoff	7.35	7.79	8.13
Sensitivity (%)	91.18 (76.32, 98,14)	96.55 (82.24, 99.91)	95.65 (78.05, 99.89)
Specificity (%)	100.00 (76.84, 98.14)	94.74 (73.97, 99.87)	80.00 (59.30, 93.17)
PPV (%)	100.00 (88.78, 100.00)	96.55 (82.24, 99.91)	81.48 (61.92, 93.70)
NPV (%)	82.35 (56.57, 96.20)	94.74 (73.97, 99.87)	95.24 (76.18, 99.88)
Accuracy (%)	93.75 (82.80, 98.69)	95.83 (85.75, 99.49)	87.50 (74.75, 95.27)

Cutoff critical cutoff value, AUC area under the receiver operating characteristic curve, PPV positive predictive value, NPV negative predictive value

AUC* adjusted for inflammation and ballooning

*S* ≥ *S1* fatty liver, *S* ≥ *S*2 moderate steatosis or higher severity, *S* = *S*3 severe steatosis

## Discussion

This study aims to demonstrate the influence of steatosis on liver stiffness measured by SWE on a rat model in the absence of fibrosis, indicating that steatosis, but not inflammation (*B* =  − 0.031, *P* = 0.920) and ballooning (*B* = 0.216, *P* = 0.458), was an independent factor affecting the mean elastic modules (*B* = 1.558, *P* < 0.001). In terms of predictive efficacy, the AUROC of the mean elastic modulus model for differentiating steatosis was > 0.90. After adjusting for inflammation and ballooning, the AUROC of the mean elastic modulus value for distinguishing the steatosis degree was also ≥ 0.92, which means SWE has excellent predictive validity for grading the steatosis stages regardless of the presence or absence of inflammation and ballooning.

SWE can quantitatively reflect tissue stiffness according to shear wave propagation in the tissue expressed as Young's modulus. Zhaoke Pi et al. [28] showed that the elasticity values µ attained by SWE in vivo had a significant correlation with liver steatosis in NAFLD in a mouse model. Grimal et al. [29] found a steadily increase of median liver stiffness in rats with NASH, measured by SWE, with exacerbation of steatosis grade. Similarly, our results also suggested that steatosis is highly associated with liver mean elastic modules. This could be explained because the presence of fat droplets within hepatocytes affects the liver structure and may alter the propagation time of vibration waves in the liver, a crucial principle of 2D-SWE.

However, several studies using SWE found no correlation between hepatic steatosis and elasticity [30–32]. Possible factors accounting for this difference could be experimental operators, steatosis type, unadjusted confounding factor, and different diets animal models. In rats fed with MCD, mainly macrovesicular steatosis developed, whereas mainly microvesicular steatosis was observed in the rats fed with a choline deficient diet [33]. Previous study had suggested that microvesicular steatosis was associated significantly with advanced fibrosis, but macrovesicular steatosis was not [34]. Therefore, different types of steatosis may affect the LSM through SWE due to the presence or absence of fibrosis. Besides, most research investigated the relationship between fibrosis with steatosis and the elastic value by subgroup analysis. A bidirectional relationship between steatosis and fibrosis is found in patients with NAFLD. Specifically, hepatic steatosis can promote fibrosis in the early stages of NAFLD, while advanced fibrosis or cirrhosis reduces the steatosis degree [35]. Consequently, it is difficult to distinguish their separate effects on LSM without adjusting for confounding factors.

In addition, multivariate regression analysis demonstrated that neither inflammation nor ballooning was independently related to liver stiffness, in accordance with previous studies [36, 37]. However, our findings differed from Sugimoto et al.’s [38] and Takeuchi et al.’s [39]. This may be due to differences in experimental subjects, viscoelasticity parameters, and the inherent limitations of non-invasive imaging methods. Interestingly, some studies in NAFLD patients pointed out that inflammation affected LSM accuracy with TE rather than with SWE [17, 31]. Accordingly, inflammation may lead to an increase in LSM with TE but not with 2D-SWE; a combination of TE and 2D-SWE could help identify suitable individuals with inflammation for participation in clinical trials.

Hepatic steatosis and lobular inflammation are strongly associated with NASH progression [40]. To diagnose NASH as early as possible, more attention should be drawn for diagnosis of steatosis. In our study, a rat model was used to limit the confounding effects of fibrosis; our results demonstrated that steatosis was an independent factor affecting elastic modules, and the AUROC of mean elastic modulus value is > 0.90. Our results indicated that LSM can provide useful information on the status of hepatic steatosis obtained by SWE with or without inflammation and ballooning. Compared to other elastography techniques, SWE can detect tissue elasticity in real time and steadily when observing two-dimensional morphology, allowing to easily select the ideal ROI for measurement of liver elasticity, thus offering more reliable estimates of elastic values.

Some advantages of our study are worth recapitulating. Firstly, at present the “gold standard” for diagnosing NAFLD is liver biopsy. However, it is not suitable for wide application due to its invasiveness, sampling error, and associated complications. Therefore, we established an animal NAFLD model to obtain imaging information and pathological data and accurately assess NAFLD histological features in SWE. Secondly, we demonstrated the influence of steatosis on liver stiffness measured by SWE by adjusting for inflammation and ballooning, and excluding fibrosis. We obtained the estimated effect through multiple regression and determined an association between elasticity and steatosis stages. Thirdly, we evaluated the predictive performance of the mean elastic modulus value for steatosis by ROC analysis, as well as the AUC value adjusted by inflammation and ballooning, and DCA and AUDCA were calculated in this study.

Several limitations should be acknowledged. Firstly, an NAFLD animal model could develop a single pathological feature like steatosis, then evaluating its impact on liver stiffness measurement, which would be preferred. With prolonged feeding of MCD, rats develop other pathological features, such as inflammation and fibrosis, that may interfere with the purpose of this study, even though we utilized the statistical approach to adjust. Secondly, to explore the association between steatosis and elastic modules, we excluded rats with fibrosis, as it has been associated with liver stiffness [16], which leads to a relatively small sample size and may induce sampling error. In addition, the proportion of rats with different degrees of steatosis was uneven, especially in the severe steatosis group. Thirdly, the animal model in our study were Sprague–Dawley rats. Rodents and humans have markedly different metabolic rates, affecting liver homeostasis [30]. Consequently, NAFLD pathogenesis may not exactly replicate in humans; so further research is required to validate and improve SWE accuracy.

In conclusion, mean elasticity was significantly associated with hepatic steatosis rather than ballooning or inflammation. The elasticity measured by SWE can reflect the grades of hepatic steatosis to a certain extent. Despite the usefulness of liver biopsy, SWE may have the feasibility to be introduced as an assistive technology for patients with NAFLD in grading steatosis. Further studies with prospective, more scientific research modalities and larger sample sizes are needed to clinically validate our results.

## Data Availability

The datasets are available from the corresponding author with reasonable request.

## Abbreviations

ARFI: Acoustic radiation force impulse
AUDCA: Area under decision curve analysis
AUROC: Area under the receiver operating characteristics curve
CAP: Controlled attenuation parameter
CI: Confidence interval
DCA: Decision curve analysis
HS: Hepatic steatosis
IQR: Interquartile range
LB: Liver biopsy
LiSA: Liver ultrasound attenuation
LSM: Liver stiffness measurement
MCD: Methionine choline efficiency
MEM: Mean elastic modulus value
MaxEM: Maximum elastic modulus value
MinEM: Minimum elastic modulus value
NAFLD: Non-alcoholic fatty liver disease
NASH: Non-alcoholic steatohepatitis
NPV: Negative predictive values
PPV: Positive predictive values
SWE: Ultrasound shear wave elastography
SD: Standard deviation
TE: Transient elastography
US: Ultrasound
